# Editorial: The pharmacotherapy of depression-searching for new mechanisms and drug interactions. Basic and clinical research, volume II

**DOI:** 10.3389/fphar.2024.1402761

**Published:** 2024-03-26

**Authors:** Katarzyna Stachowicz, Magdalena Sowa-Kućma, Anna Tabecka-Łonczyńska

**Affiliations:** ^1^ Department of Neurobiology, Maj Institute of Pharmacology, Polish Academy of Sciences, Kraków, Poland; ^2^ Department of Human Physiology, Institute of Medical Sciences, Medical College of Rzeszow University, Rzeszow, Poland; ^3^ Department of Biotechnology and Cell Biology, Medical College, University of Information Technology and Management in Rzeszow, Rzeszow, Poland

**Keywords:** new mechanisms, new drugs interactions, side effects, depression, pharmacotherapy

The second edition of the e-book “*The pharmacotherapy of depression-searching for new mechanisms and drug interactions*” includes research and review papers.

Three Reviews present the mechanisms of autophagy, ferroptosis, and mitophagy as a new strategy for treating depression (Lv et al.; Zhang et al.; Xu et al., respectively).

Autophagy is a crucial mechanism of intracellular degradation, while ferroptosis is a form of cell death caused by excessive accumulation of iron-dependent lipid peroxides. The importance of these two cellular processes in developing and treating depression has been extensively discussed by Lv et al. and Zhang et al., respectively. The authors presented basic information about autophagy and ferroptosis and the possible connections between these processes and depression. Furthermore, the importance of these processes in response to classical antidepressants (e.g., fluoxetine, agomelatine, ketamine) and active ingredients derived from Traditional Chinese Medicine (TCM) have been widely discussed. Various strengths, limitations, and research prospects targeting autophagy and ferroptosis in depression have been highlighted.

Mitophagy is the process of removing excess or dysfunctional mitochondria. Disturbances in the proper course of mitophagy are associated with the progression of depression. To date, presented results have allowed the creation of new pharmacological strategies that may stimulate mitophagy and, at the same time, alleviate depression. It was observed that several antidepressants, such as fluoxetine and ketamine, as well as compounds used in TCM (baicalin), brought good therapeutic results related to the induction of mitophagy (Xu et al.). Further comprehensive research, therefore, brings great hope for the use of innovative therapies to treat depression.

Also, new potential antidepressants and their molecular targets are presented in this e-book. Ye et al., using a maternal separation with an early weaning model [early life stress (ELS) model - the leading risk factor for depression in teenagers), demonstrated the antidepressant-like effect of the Si-Ni-San formula (SNS - a fundamental prescription for treating depression in TCM) in three primary tests (sucrose preference test, forced swim test, tail suspension test) for examining the antidepressant activity of compounds in mice. The observed behavioral alterations were strongly related to RAS-related C3 Botulinum Toxin Substrate 1 (Rac1) activity and associated spine plasticity in the NAc (Ye et al.).

Substantial evidence for the effectiveness and safety of quinolone in the treatment of major depressive disorder (MDD) is provided by a systematic review by Wang et al. This neuroactive steroid (NAS) and GABA-A receptor-positive allosteric modulator (PAM) was approved by the FDA as the first oral treatment for postpartum depression, and its effectiveness in this disorder is well documented. The presented meta-analysis, including 4 studies involving a total of 1,454 patients, shows fate of quinolone research in MDD treatment (Xu et al.). Furthermore, Dudek et al. showed that the efficacy of trazodone (a serotonin 5-HT2 receptor antagonist and reuptake inhibitor) in patients with MDD administered in an extended-release form was comparable to or even superior to SSRIs.

However, it is essential to analyze adverse events associated with the use of antidepressants. Here, adverse events associated with the use of antidepressants along with adaptogens were described (Siwek et al.). Nearly 9% of adverse events are due to the use of antidepressants with other medications, so following the author’s suggestion, the clinic should consider better monitoring of the medications patients are taking to avoid side effects and pharmacological interactions (Siwek et al.). Successively, sertraline used as an antidepressant was found to be involved in increasing thioflavin-S and Congo red deposition in APPswe/PSEN1dE9 mice (Liao et al.). The changes were connected with hippocampus gliosis and decreased recognition index in APP/PSEN1 mice (Liao et al.). These studies indicate the need to monitor the use of sertraline in patients with Alzheimer’s disease (AD) and co-morbid depression.

Increasingly, scientists are paying detailed attention to Chinese medicine. It is also used in the treatment of depression. Interestingly, the effectiveness and safety of Chinese herbal medicines in antidepressant therapy were assessed using meta-analysis. The results were compared with standard pharmacotherapies by searching multiple databases with research results. As shown, traditional medicines such as fluoxetine, escitalopram, amitriptyline, sertraline, flupentixol, melintracene, and venlafaxine are less effective and have a higher risk of adverse symptoms than Chinese herbal medicines. Therefore, it seems that the potential of Chinese herbs may be an effective alternative to classic therapeutic procedures in the treatment of depression. However, long-term studies in patients are needed to confirm effectiveness and safety (Chun et al.).

